# In-hospital mortality trends among patients with idiopathic pulmonary fibrosis in the United States between 2013-2017: a comparison of academic and non-academic programs

**DOI:** 10.1186/s12890-020-01328-y

**Published:** 2020-11-07

**Authors:** Shehabaldin Alqalyoobi, Evans R. Fernández Pérez, Justin M. Oldham

**Affiliations:** 1 Division of Pulmonary, Critical Care and Sleep Medicine, Department of Medicine, East Carolina University-Brody School of Medicine, Greenville, North Carolina USA; 2 Present address: Internal Medicine - Pulmonary, Critical Care, and Sleep Medicine, Brody School of Medicine, Mail Stop 628, 3E-149, Greenville, NC 27834 USA; 3 Division of Pulmonary, Critical Care and Sleep Medicine, National Jewish Health, Denver, CO USA; 4 Department of Internal Medicine; Division of Pulmonary, Critical Care and Sleep Medicine, University of California at Davis, Sacramento, CA USA

**Keywords:** Idiopathic pulmonary fibrosis, Mortality, Academic hospital, Respiratory failure, Mechanical ventilation

## Abstract

**Background:**

Idiopathic pulmonary fibrosis (IPF) is a devastating condition characterized by progressive lung function decline and early mortality. While early accurate diagnosis is essential for IPF treatment, data evaluating the impact of hospital academic status on IPF-related mortality remains limited. Here we examined in-hospital mortality trends for patients with IPF from 2013 to 2017. We hypothesized that in-hospital IPF mortality would be influenced by hospital academic setting.

**Methods:**

Hospitalization data was extracted from the National Inpatient Sample (NIS) for subjects with an international classification of disease code for IPF. In-hospital mortality stratified by hospital setting (academic versus non-academic) was the primary outcome of interest, with secondary analyses performed for subgroups with and without respiratory failure and requiring mechanical ventilation. Predictors of mortality were then assessed.

**Results:**

Among 93,680 patients with IPF requiring hospitalization, 58,450 (62.4%) were admitted to academic institutions. In-hospital mortality decreased significantly in those admitted to an academic hospital (*p* < 0.001) but remained unchanged in patients admitted to a non-academic hospital. A plateau in-hospital mortality was observed among all hospitalized patients (*p* = 0.12), with a significant decrease observed for patients with admitted respiratory failure (*p* < 0.001) and those placed on mechanic ventilation (*p* < 0.001).

**Conclusion:**

In-hospital mortality decreased significantly for patients with IPF admitted to an academic hospital, suggesting that management strategies may differ by hospital setting. Mortality among those with respiratory failure and those requiring mechanical ventilation has dropped significantly. Our findings may underscore the importance of promoting early referral to an academic institution and adherence to international treatment guidelines.

**Supplementary Information:**

The online version contains supplementary material available at 10.1186/s12890-020-01328-y.

## Background

Idiopathic pulmonary fibrosis (IPF) is a progressive, fatal lung disease characterized by high mortality and unpredictable natural history [1]. An uncommon, but deadly complication of IPF is acute exacerbation, which often results in respiratory failure (RF) and requires hospitalization [2]. While acute exacerbations of IPF are difficult to characterize due to lack of an international classification of disease (ICD) code, IPF related hospitalizations have been suggested as a clinically meaningful endpoint [2–6]. This is supported by prior work showing respiratory hospitalization is associated with increased short-term mortality risk in patients with IPF [7]. Therapeutic advances over the last decade have led to the first FDA approved therapies for the treatment of IPF [8]. Anti-fibrotic therapies nintedanib and pirfenidone have both been shown to slow lung function decline in patients with IPF [4, 9] and recent analyses suggest they may improve survival, [10–12] reduce hospitalization, [4, 10] and exacerbation risk [4, 13].

While early accurate diagnosis is essential for IPF treatment, significant disagreements between community-based physicians and academic-based physicians in the diagnosis of interstitial lung diseases have been described before [14]. Besides, delayed access to academic hospitals has been associated with decreased survival in IPF patients [15]. One mechanism by which these delays may reduce survival stem from the delay in receiving an accurate diagnosis and initiation of ineffective or harmful interventions [15]. Whether these observations result in differential in-hospital mortality in patients with IPF remains unknown.

In this investigation, we used the National Inpatient Sample (NIS) database to assess in-hospital mortality trends for patients with IPF from 2013 to 2017. We hypothesized that in-hospital IPF mortality would be influenced by hospital setting (academic versus non-academic). We also assessed whether these trends were influenced by presence of respiratory failure and use of mechanic ventilation. Finally, we assessed whether clinical characteristics at the time of hospitalization were associated with in-hospital mortality.

## Methods

### Data source

The study was conducted using the NIS database for years 2013–2017. The Agency for Healthcare Research and Quality developed this data for healthcare cost and utilization project [16]. All patient data contained in these database files have been deidentified and are on public record; therefore, Institutional Review Board approval for this study was not required. The NIS assesses the data quality periodically to ensure its internal validity [17]. It contains more than 100 clinical data elements from ~ 7 million unweighted admissions (weighted to ~ 35 million admissions) annually, representing 20% of hospital admission in the United states. It uses ICD-9 codes through September 2015 and ICD-10 codes thereafter.

### Study population

We identified records for subjects ≥50 years with any idiopathic pulmonary fibrosis (IPF) diagnosis codes (ICD-9, 516.31; ICD-10, J84.112). We excluded any patients who had concomitant diagnosis codes for connective tissue diseases (CTD), hypersensitivity pneumonitis, other exposure-related diseases (drugs and radiation) or lung transplant (Tables E1, E2, E3, E4). We also excluded patients with two or more idiopathic interstitial pneumonias diagnoses or any IPF diagnosis along with any code related to non-specific fibrosis, including those used in prior studies of other conditions, such as post-inflammatory fibrosis (ICD-9515 and ICD-10 J84.10) (Table E1) [18].

### Patient and hospital characteristics

Baseline patients demographics (age, race, and sex) and relevant comorbidities (smoking, chronic obstructive lung disease (COPD), asthma, respiratory failure (RF) (acute, chronic, acute on chronic or non-specified), obstructive sleep apnea (OSA), gastroesophageal reflux disease (GERD), low body mass index (BMI < 20), frailty, pneumonia, congestive heart failure (CHF), obesity, renal failure, liver disease, diabetes mellitus (DM), hypothyroidism, new pulmonary embolism (PE) and cancer (Solid and Metastatic)) were extracted (Table E2, E3, E4). Other inpatient diagnoses and procedures using ICD-9 and ICD-10 codes (Table E3), Clinical Classifications Software codes (Table E4), and Elixhauser comorbidities (Table E2) [19–22]. and hospital size and setting for each hospitalization were also extracted.

### Outcomes measured

The primary outcome assessed was in-hospital mortality, defined as death during the hospitalization encounter, stratified by academic hospital status (academic versus non-academic). The hospital is identified as an academic center in the NIS database if it has one or more Accreditation Council for Graduate Medical Education (ACGME) approved residency programs, is a member of the Council of Teaching Hospitals (COTH) or has a ratio of full-time equivalent interns and residents to beds of .25 or higher. Non-academic hospitals did not meet the above criteria or located in rural areas. Secondary analyses were performed in the following subgroups (1) those hospitalized with and without respiratory failure and (2) those requiring mechanical ventilation. A list of the ICD-9 and ICD-10 codes for the secondary outcome is included in supplementary materials (Tables E1, E2, E3, E4).

### Statistical analysis

Continuous variables are reported as mean with standard deviation (SD) and compared using Student’s t-test. Categorical values are reported as count and percentage compared using the Chi-square test. A Cochran-Armitage test of trend was used to assess linear trend in mortality. Univariable and multivariable logistic regression was performed to identify independent predictors of in-hospital mortality. Variables were selected based on previous studies [23–25]. A Bonferroni correction was applied using all eighteen terms in the multivariable logistic regression model resulting in statistical significance being accepted when *p* < 0.003 [26]. The area under the curve of the receiver operator characteristic was calculated to assess risk explanation. Statistical significance was defined as *p* < 0.05 unless stated otherwise. Statistical analyses were performed using SPSS (IBM SPSS Statistics for MAC, Version 26.0; Armonk, New York: IBM Corp Released 2019) and SAS software, university edition (SAS Institute, Inc., Cary, NC, USA).

## Results

### Population characteristics

**Fig. 1:**
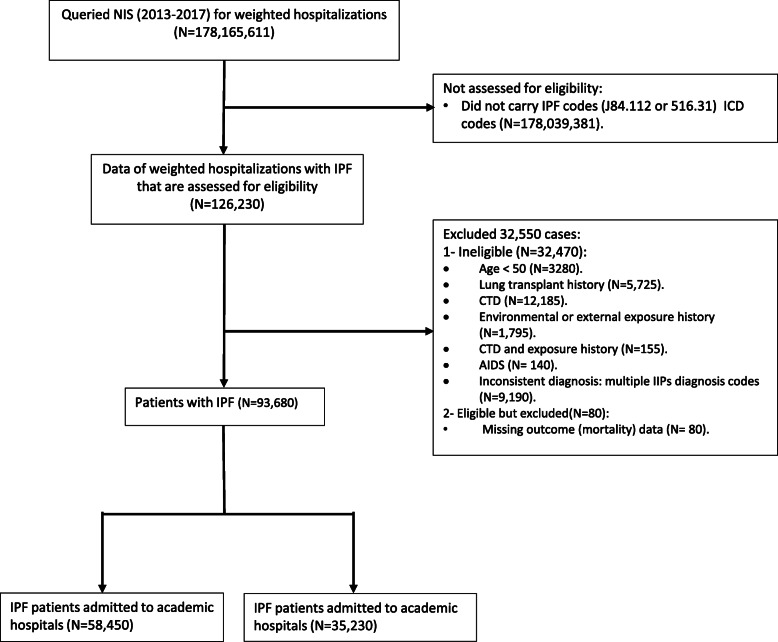
STROBE diagram (AIDS: acquired immunodeficiency syndrome; CTD: connective tissue disease; ICD: international classification of diseases; IPF: idiopathic pulmonary fibrosis; NIS: national inpatient sample)

**Fig. 2:**
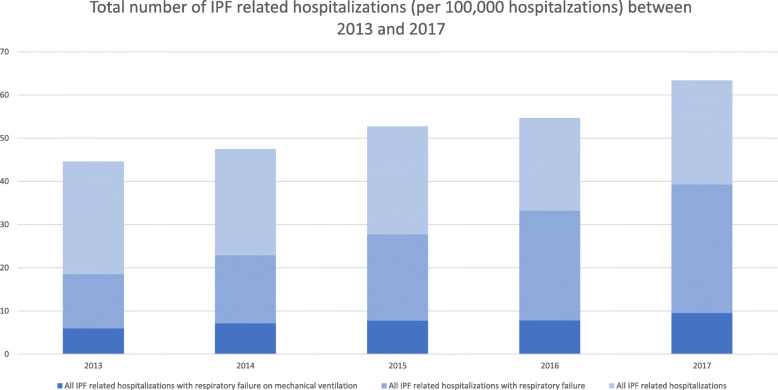
Temporal trends in IPF related hospitalizations (per 100,000 hospitalizations)

From 2013 to 2017, 126,230 weighted records with IPF were identified. Of these, 93,680 hospitalizations met inclusion criteria, including 58,450 (62.4%) admitted to an academic hospital (Fig. 1). We report an incidence of all-cause hospitalizations among patients with IPF of 44.6 per 100,000 hospitalizations for 2013 and 63.4 per 100,000 hospitalizations for 2017 which increased by 42.2% over the five years (Fig. 2). In IPF patients hospitalized with respiratory failure, the incidence increased by 2 folds as it was 18.6 per 100,000 hospitalizations for 2013 and 39.3 per 100,000 hospitalizations for 2017. While the incidence for IPF patients with respiratory failure requiring mechanical ventilation increased by 61% from was 5.9 per 100,000 hospitalizations for 2013 to 9.5 per 100,000 hospitalizations for 2017 (Fig. 2). Baseline characteristics stratified by the hospital academic status are presented in (Table 1**)**. Those who are admitted to an academic institution had significantly more males and Black patients with a higher proportion of respiratory failure, obstructive sleep apnea, gastroesophageal reflux disease, low body mass index, obesity, new pulmonary embolism, pulmonary circulation disorders, and supplemental oxygen therapy use. The same group also had more patients who underwent bronchoscopy, required mechanical ventilation, and were admitted for a longer period. Individuals who are admitted to non-academic hospitals were significantly older with more White and Hispanic patients and with a higher proportion of elective admissions, chronic obstructive pulmonary disease, pneumonia, and diabetes mellitus.

**Table 1:** Clinical characteristic between IPF cases admitted to academic vs non-academic hospitals, 2013–2017

Variables	IPF hospitalizations in academic institutions (*n* = 58,450)	IPF hospitalizations in non-academic institutions (*n* = 35,230)	***P***-Value
Age, mean ± SD	74.5 ± 10.1	75.7 ± 10	< 0.001
Female, *n* (%)	25,035 (42.8)	15,785 (44.8)	< 0.001
Length of stay, mean ± SD	6.6 ± 7	5.7 ± 5	< 0.001
Race			< 0.001
White, *n* (%)	42,330 (76.1)	27,950 (82.8)	
Black, *n* (%)	4785 (8.6)	1715 (5.1)	
Hispanic, *n* (%)	5130 (9.2)	2495 (7.4)	
Ever smoker, *n* (%)	25,760 (44.1)	14,630 (41.5)	0.1
Elective admission, *n* (%)	6040 (10.4)	3750 (10.7)	< 0.001
Respiratory failure, *n* (%)	31,970 (54.7)	18,585 (52.8)	< 0.001
Mechanically ventilated IPF patients, *n* (%)	9900 (16.9)	5135 (14.6)	< 0.001
Bronchoscopy, *n* (%)	4935 (8.4)	1825 (5.2)	< 0.001
Dependence on long-term Oxygen, *n* (%)	21,320 (36.5)	11,235 (31.9)	< 0.001
Co-morbidities			
Chronic obstructive lung disease, *n* (%)	6010 (10.3)	6245 (17.7)	< 0.001
Obstructive sleep apnea, *n* (%)	8530 (14.6)	4035 (11.5)	< 0.001
Gastroesophageal reflux disorder, *n* (%)	19,785 (33.8)	10,435 (29.6)	< 0.001
Pneumonia, *n* (%)	17,440 (29.8)	11,970 (34)	< 0.001
Low body mass index, *n* (%)	2190 (3.7)	985 (2.8)	< 0.001
Obesity, *n* (%)	7265 (12.4)	4015 (11.4)	< 0.001
Frailty, *n* (%)	190 (0.3)	105 (0.3)	0.48
Diabetes mellitus, *n* (%)	19,430 (33.2)	11,720 (33.3)	0.006
New pulmonary embolism, *n* (%)	1570 (2.7)	780 (2.2)	< 0.001
Asthma, *n* (%)	4240 (7.3)	2500 (7.1)	0.37
Congestive heart failure, *n* (%)	15,580 (26.7)	9535 (27.1)	0.17
Pulmonary circulation disease, *n* (%)	6640 (11.4)	3790 (10.8)	0.005
Chronic renal disease, *n* (%)	12,215 (20.9)	6595 (18.7)	0.69
Liver disease, *n* (%)	2150 (3.7)	1045 (3)	0.74
Solid tumor w/o metastasis, *n* (%)	1755 (3)	1040 (3)	0.66
Metastatic cancer, *n* (%)	1000 (1.7)	590 (1.7)	0.68
Hypothyroidism, *n* (%)	11,460 (19.6)	7020 (19.9)	0.23
Elixhauser sum of conditions			
Mean ± SD	4 ± 2	4 ± 2	0.22

### IPF in-hospital mortality trends

**Fig. 3:**
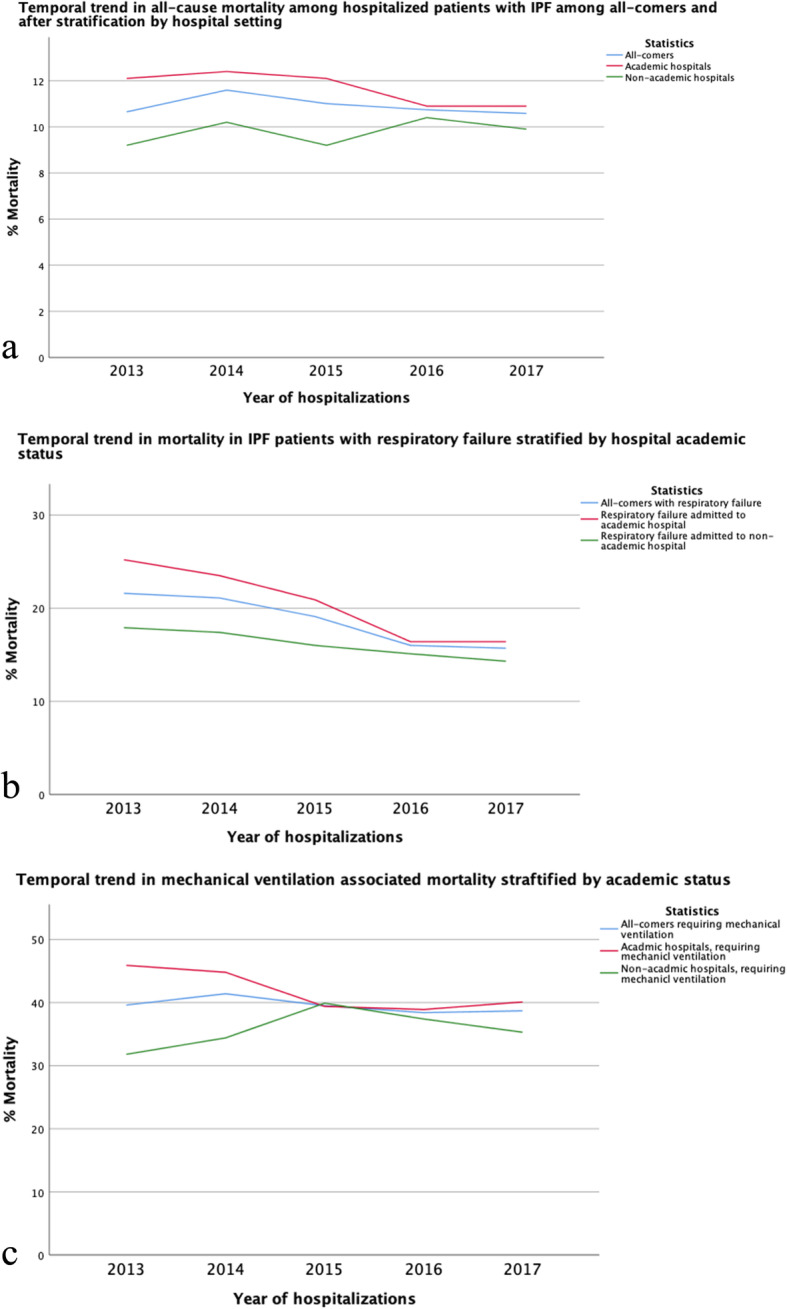
**a** Temporal trend in all-cause mortality among hospitalized patients with IPF among all-comers and after stratification by hospital setting (IPF: idiopathic pulmonary fibrosis). **b** Temporal trend in mortality in IPF patients with respiratory failure stratified by hospital academic status. **c** Temporal trends in mechanical ventilation associated mortality stratified by academic status of the hospital

**Fig. 4:**
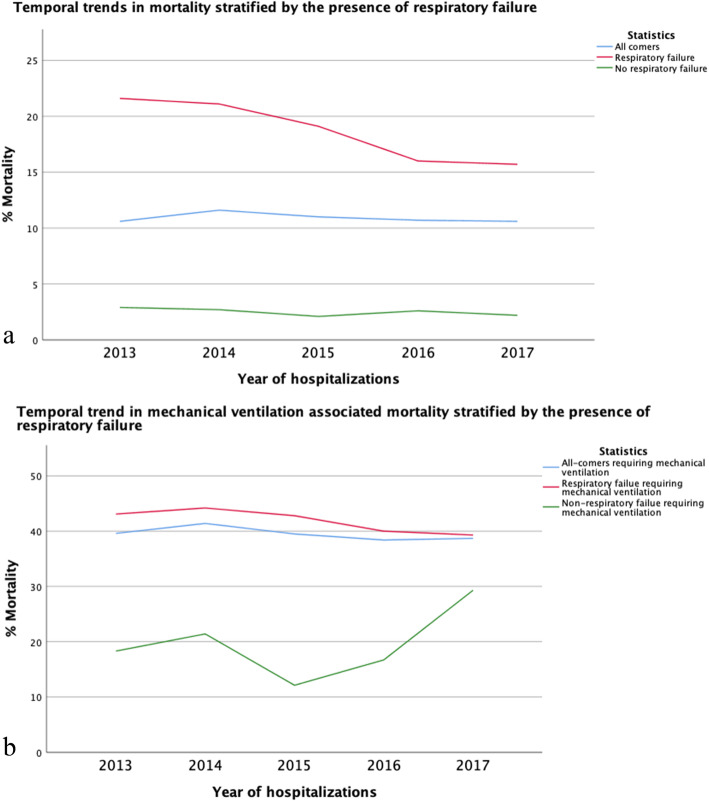
**a** Temporal trends in mortality stratified by the presence of respiratory failure. **b** Temporal trends in mechanical ventilation associated mortality stratified by presence of respiratory failure

Total hospitalizations, hospitalizations with respiratory failure and hospitalizations with respiratory failure requiring mechanical ventilation are shown in Fig. 2. Mean in-hospital mortality for years 2013–2017 was 10.9%, which showed a non-statistically significant decline over time (*p* = 0.12) (Fig. 3**a**) **(Table E**5**a)**. While mortality for patients admitted to non-academic hospitals was < 10%, mean in-hospital mortality for patients admitted to academic institution was 11.6%, which declined significantly over time (*p* < 0.001) (Fig. 3 a) **(Table E**5**b)**. The in-hospital mortality did not change in those admitted to non-academic institution **(**Fig. 3 a) **(Table E**5**b)**. In-hospital mortality among patients admitted with respiratory failure also decreased among patients admitted to an academic institution (*p* < 0.001) and decreased in those admitted to a non-academic institution (*p* < 0.001) (Fig. 3 b) **(Table E**5**b)**. Among patients requiring mechanical ventilation, in-hospital mortality significantly declined in patients admitted to an academic institution (*p* < 0.001) but increased in patients hospitalized in non-academic institutions (*P* = 0.03) (Fig. 3 c) **(Table E**5**b)**. While mortality for patients admitted without respiratory failure was < 5%, mean in-hospital mortality for patients admitted with respiratory failure was 18.1%, which declined significantly over time (*p* < 0.001) (Fig. 4 a) **(Table E**5**c)**. When assessing patients who required mechanical ventilation, those without respiratory failure had no change in mortality (*p* = 0.1) over time, while those admitted with respiratory failure showed a significant decline in mortality over time (*p* < 0.001) (Fig. 4 b) **(Table E**5**d)**. When stratifying by age, mortality was similar across age groups, except IPF patients in age group (50–59) years old where it declined significantly (*P* = 0.001) **(Table E**5**e) (Fig. E****1****)**. In addition, mechanical ventilation therapy declined significantly in IPF patients with and without respiratory failure (*P* < 0.001) **(Table E**5**f) (Fig. E**2**)**.

### Predictors of in-hospital IPF mortality

In unadjusted logistic regression, predictors of mortality included admission to an academic hospital, respiratory failure, receiving mechanical ventilation therapy, bronchoscopy, frailty, low body mass index, pneumonia, new pulmonary embolism, and dependence on long-term oxygen therapy. Based on Bonferroni correction assessment, elective admission (OR 1.28, 95% CI 1.16–1.4), admission to an academic hospital (OR 1.14, 95% CI 1.09–1.2), respiratory failure (OR 4.77, 95% CI 4.34–5.13), receiving mechanical ventilation therapy (OR 7.26, 95% CI 6.9–7.64), bronchoscopy (OR 1.23, 95% CI 1.13–1.33),) low body mass index (OR 1.51, 95% CI 1.34–1.69), pneumonia (OR 1.38, 95% CI 1.31–1.45), and new pulmonary embolism (OR 1.83, 95% CI 1.62–2.08) were found to be independent predictors of mortality in the multivariable logistic regression model (Table 2).

**Table 2:** Conditions and interventions predicting in-hospital mortality in patients with IPF patients

Risk factors	Unadjusted			Adjusted Model*		
	OR	***p***-value	95%CI	OR	***p***-value**	95%CI
Age	0.995	< 0.001	(0.993–0.997)	1.007	< 0.001	(1.004–1.009)
Female	0.72	< 0.001	(0.7–0.76)	0.77	< 0.001	(0.73–0.81)
Race***:						
Black	0.83	< 0.001	(0.76–0.91)	0.69	< 0.001	(0.62–0.76)
Hispanic	0.9	0.008	(0.83–0.97)	0.67	< 0.001	(0.62–0.74)
Ever Smoker	0.83	< 0.001	(0.8–0.86)	0.79	< 0.001	(0.75–0.83)
Elective admission	0.63	< 0.001	(0.58–0.68)	1.28	< 0.001	(1.16–1.4)
Academic hospital	1.21	< 0.001	(1.16–1.27)	1.14	< 0.001	(1.09–1.2)
Any respiratory failure	8.67	< 0.001	(8.13–9.25)	4.77	< 0.001	(4.34–5.13)
Mechanical ventilation therapy	11.36	< 0.001	(10.86–11.88)	7.26	< 0.001	(6.9–7.64)
Bronchoscopy	2.25	< 0.001	(2.11–2.39)	1.23	< 0.001	(1.13–1.33)
Gastroesophageal reflux disorder	0.82	< 0.001	(0.78–0.86)	0.84	< 0.001	(0.8–0.89)
Obstructive sleep apnea	0.78	< 0.001	(0.74–0.84)	0.62	< 0.001	(0.58–0.67)
Diabetes mellitus	0.94	0.008	(0.9–0.99)	0.91	0.001	(0.87–0.96)
Frailty	1.67	0.001	(1.23–2.27)	1.48	0.03	(1.04–2.1)
Low body mass index	1.65	< 0.001	(1.5–1.82)	1.51	< 0.001	(1.34–1.69)
Obesity	0.81	< 0.001	(0.76–0.87)	0.77	< 0.001	(0.71–0.84)
Pneumonia	2.31	< 0.001	(2.22–2.41)	1.38	< 0.001	(1.31–1.45)
New pulmonary embolism	2.18	< 0.001	(1.97–2.42)	1.83	< 0.001	(1.62–2.08)
Dependence on long-term Oxygen	1.23	< 0.001	(1.18–1.28)	0.91	< 0.001	(0.87–0.96)

*Adjusted for all variables mentioned in this table

** Statistically significant *P*-value cutoff after Bonferroni correction is (*p* < 0.003)

*** Compared to white

The logistic regression model was statistically significant, χ2 = 14,153.7, *p* < 0.001. The model explained 29.7% (R2) of the variance in mortality and correctly classified 89.4% of cases. Sensitivity was 16.1%, specificity was 98.3%, positive predictive value was 53% and negative predictive value was 90.7%. The area under the ROC curve was 0.835 (95% CI, 0.831 to 0.839), which is an excellent level of discrimination **(Figs. E**3**)**

## Discussion

In this study, we examined in-hospital mortality trends in patients with IPF from 2013 and 2017, which spanned the years immediately preceding and after the approval of anti-fibrotic therapy to treat IPF. We found that while in-hospital mortality was 10.9%, mortality was higher among patients admitted to academic hospital (11.6%) and even significantly higher in those with respiratory failure (20.5%), and those requiring mechanical ventilation (41.8%) who are admitted to academic centers. While in-hospital mortality did not significantly change over time for all-comers, mortality did significantly decrease in patients admitted to academic hospitals, including those with respiratory failure and those requiring mechanical ventilation. We reported no significant change in all-cause mortality in patients admitted to a non-academic institution. While respiratory failure associated mortality decreased significantly in IPF patients admitted to non-academic centers, mechanical ventilation-associated mortality increased significantly in this group. Subgroup analysis showed that mortality did significantly decrease in patients admitted with respiratory failure and in those requiring mechanical ventilation. These observations might suggest that the early referral to academic centers may reduce IPF mortality.

Our data demonstrate increasing all-cause hospitalizations for patients with IPF from 2013 to 2017, which may reflect previously reported increasing incidence and prevalence of IPF in the US [27, 28]. Despite this increase in hospitalizations, our data suggest a relatively static in-hospital mortality for patients admitted during this timeframe. These findings are supported by others using the NIS dataset, who reported similar all-cause mortality in patients with IPF admitted to the hospital 2006 to 2012 [29] and others using a similar dataset, who reported IPF mortality during index admission from 2011 and 2014 to be 10.3% [30]. These findings stand in contrast to those published using the online CDC national death certificate database, which showed IPF-related mortality to be increasing over this timeframe [31, 32]. Besides, others reported decline in IPF all-cause mortality and hospitalizations using NIS dataset [33]. With different case finding methodologies employed by each study, these observations highlight the difficulties with capturing accurate IPF data using claims databases.

During the study period, when IPF hospitalizations were stratified by hospital academic status, we found a significant decline in all-cause mortality, respiratory failure associated mortality and mechanical ventilation associated mortality in IPF patients admitted to teaching hospitals. Interestingly, we found a significant increase in mechanical ventilation associated mortality in IPF patients hospitalized in a non-academic institution. No significant changes in all-cause mortality in IPF patient admitted to a non-academic hospital while respiratory associated mortality decreased significantly in the same group. The reasons underpinning these observations remain unclear but may suggest a stronger adherence to 2015 IPF treatment guidelines at academic centers [34]. Besides, others have shown that significant disagreement exists in the diagnosis of ILD between community-based physicians and academic physicians [14]. We also hypothesize that formal multidisciplinary discussion for IPF diagnosis would be conducted in academic centers and unlikely to be performed in non-academic institutions [35]. Early access to lung transplant service and anti-fibrotic therapy might explain this observation as well. Others have shown that early referral of IPF patients to tertiary care centers is associated with reduced mortality, supporting an added benefit provided at these centers [15]. We also found that admissions to the academic centers were associated with higher mortality risk, which is similar to previous studies [36]. This might reflect more advanced diseases in IPF patients referred to the academic centers as they included patients referred for lung transplant evaluation and other advanced therapeutics. We also speculate that academic centers receive sicker IPF patients as admission to academic centers is described as an independent risk factor for receiving mechanical ventilation therapy [36]. However, our assumption is limited by our data type and documentation bias.

We observed a significant decline in respiratory failure-associated mortality over the years assessed. Additionally, despite the plateau in mechanical ventilation associated mortality in the whole cohort, mechanical ventilation therapy and mechanical ventilation associated mortality in the respiratory failure group declined significantly. The mortality rate in IPF patients with respiratory failure receiving mechanical ventilation therapy has been reported to range from 50 to 90% [2, 29, 36]. Others reported mortality of 55.7% in intubated IPF patients between 2009 and 2011 using a different case definition for IPF codes (ICD9, 516.3). [36]Another study showed declining mortality between 2006 and 2013 from 58.4 to 49.3% using the same database, but different case definition [29]. In our cohort, the decline in the respiratory failure associated mortality, mechanical ventilated associated mortality and mechanical ventilation therapy is likely multifactorial and might reflect evolving and increased adherence to evidence-based pharmacological and non-pharmacological management strategies [34].

The influence of comorbid conditions and interventions on IPF mortality has been increasingly studied over the last decade [23]. Our study supports the findings of others who have shown age, sex [30, 37–39], race and smoking history [37, 38, 40] to confer differential mortality risk. Respiratory failure and need for mechanical ventilation therapy were the strongest predictors of in-hospital mortality, which supports prior findings [7, 30]. It is unclear why elective admission has been associated with increased mortality. One theory would be that elective admissions might be related to referrals from non-academic hospitals or urgent admissions from the outpatient clinic. In our assessment of comorbid conditions, our findings supported the work of other showing mortality risk to be increased in patients with pneumonia, [36, 41] low body mass index, [42] and thromboembolic disease, [43]. We found that those with concurrent obesity, GERD, diabetes and sleep apnea had lower mortality risk, which adds to mixed results with these conditions [24, 25, 44–46]. Finally, long term oxygen therapy was associated with decreased in-hospital mortality in our analysis. It is unclear if this is a true effect or this result is confounded by the presence of other diseases in which oxygen use is associated with improved survival. Further studies need to evaluate the impact of long-term oxygen use on IPF patients’ survival.

This study has several limitations. First, we used an administrative database, in which coding and documentation errors are inherent limitations. In attempts to mitigate the potential errors, multiple internal quality control measures are conducted to validate the NIS [17]. In addition, ICD-coding for IPF patients is challenging, given the complexity of the IPF diagnosis process, and might be another source of error. Therefore, we adopted a conservative approach which may result in missed cases and lower sensitivity at the expense of increased specificity. We included only patients with IPF specific codes (ICD-9, 516.31; ICD-10, J84.112), and we did not include less precise codes (ICD9, 516.3 or 515; ICD10, J84.1 or J84.9) used in previous studies [29–31, 36].. Vu et al. [18] showed in a USA population-based study that only 4% of patients with IPF ICD9 code 515 had definite or probable IPF by 2018 Fleischner criteria. A Finnish study showed that 20–30% of patients with ICD10 codes J84.1 or J84.9 met IPF criteria [47]. We also excluded any patients who had a concomitant diagnosis of environmental exposure or CTD [48]. Second, our study is a retrospective observational study based on discharge data, and it is liable to selection bias and can only assess association and not assess causation. Finally, the population studied in this period is heterogenous as it includes patients treated with and without antifibrotic therapy. We were not able to retrieve antifibrotic treatment data for our analysis, therefore our results may or may not reflect the impact of the 2014 approval of anti-fibrotic therapy for the treatment of IPF. However, our data do potentially support the work of others, who have shown antifibrotic therapy to be associated with decreased mortality, respiratory hospitalization and AE-IPF [6, 10, 49].

## Conclusion

This observational analysis from a nationally representative inpatient sample from 2013 to 2017 showed a decline in all-cause mortality, respiratory associated mortality and mechanical ventilation associated mortality in IPF patients admitted to academic hospitals, while mechanical ventilation associated mortality increased in those admitted to non-academic hospitals. We also found that respiratory failure associated mortality and mechanical ventilation associated mortality decreased in IPF patients over the same period. Our findings may underscore the importance of promoting timely diagnosis, early referral to an academic institution and adherence to international treatment guidelines. It is not clear why despite the significant decline in overall mortality, admission to the academic centers remain an independent predictor of mortality. Further research is needed to elucidate the factors driving these findings.

## Supplementary Information

**Additional file 1.**

**Additional file 2 **: **Figure E1**. Temporal trends of all-cause mortality stratified by age group. **Figure E2**. Temporal trends of MV therapy rate stratified by presence of respiratory failure. **Figure E3**. Receiver operator curve for the regression model.

## Data Availability

The datasets generated and/or analyzed during the current study are available in the Healthcare Cost and Utilization Project (HCUP) repository (https://www.hcup-us.ahrq.gov/db/nation/nis/nisdbdocumentation.jsp).

## Abbreviations

IPF: Idiopathic pulmonary fibrosis
NIS: National Inpatient Sample
ICD: International classification of disease
CTD: Connective tissue diseases
COPD: Chronic obstructive lung disease
RF: Respiratory failure
OSA: Obstructive sleep apnea
GERD: Gastroesophageal reflux disease
BMI: Body mass index
CHF: Congestive heart failure
DM: Diabetes mellitus
PE: Pulmonary embolism
ACGME: Accreditation Council for Graduate Medical Education
COTH: Council of Teaching Hospitals

## References

[1] Sgalla G, Biffi A, Richeldi L (2016). Idiopathic pulmonary fibrosis: diagnosis, epidemiology and natural history. Respirology.

[2] Collard HR, Ryerson CJ, Corte TJ, Jenkins G, Kondoh Y, Lederer DJ, Lee JS, Maher TM, Wells AU, Antoniou KM (2016). Acute exacerbation of idiopathic pulmonary fibrosis. An international working group report. Am J Respir Crit Care Med.

[3] Raghu G, Collard HR, Anstrom KJ, Flaherty KR, Fleming TR, King TE, Martinez FJ, Brown KK (2012). Idiopathic pulmonary fibrosis: clinically meaningful primary endpoints in phase 3 clinical trials. Am J Respir Crit Care Med.

[4] Ley B, Swigris J, Day BM, Stauffer JL, Raimundo K, Chou W, Collard HR (2017). Pirfenidone reduces respiratory-related hospitalizations in idiopathic pulmonary fibrosis. Am J Respir Crit Care Med.

[5] Collard HR, Brown KK, Martinez FJ, Raghu G, Roberts RS, Anstrom KJ (2014). Study design implications of death and hospitalization as end points in idiopathic pulmonary fibrosis. Chest.

[6] Nathan SD, Albera C, Bradford WZ, Costabel U, Glaspole I, Glassberg MK, Kardatzke DR, Daigl M, Kirchgaessler KU, Lancaster LH (2017). Effect of pirfenidone on mortality: pooled analyses and meta-analyses of clinical trials in idiopathic pulmonary fibrosis. Lancet Respir Med.

[7] Durheim MT, Collard HR, Roberts RS, Brown KK, Flaherty KR, King TE, Palmer SM, Raghu G, Snyder LD, Anstrom KJ (2015). Association of hospital admission and forced vital capacity endpoints with survival in patients with idiopathic pulmonary fibrosis: analysis of a pooled cohort from three clinical trials. Lancet Respir Med.

[8] Raghu G, Selman M (2015). Nintedanib and pirfenidone. New antifibrotic treatments indicated for idiopathic pulmonary fibrosis offer hopes and raises questions. Am J Respir Crit Care Med.

[9] Collard HR, Richeldi L, Kim DS, Taniguchi H, Tschoepe I, Luisetti M, Roman J, Tino G, Schlenker-Herceg R, Hallmann C, du Bois RM. Acute exacerbations in the INPULSIS trials of nintedanib in idiopathic pulmonary fibrosis. Eur Respir J. 2017;49(5):1601339. 10.1183/13993003.01339-2016 28526798

[10] Dempsey TM, Sangaralingham LR, Yao X, Sanghavi D, Shah ND, Limper AH (2019). Clinical effectiveness of Antifibrotic medications for idiopathic pulmonary fibrosis. Am J Respir Crit Care Med.

[11] Fisher M, Nathan SD, Hill C, Marshall J, Dejonckheere F, Thuresson PO, Maher TM: Predicting Life Expectancy for Pirfenidone in Idiopathic Pulmonary Fibrosis. J Manag Care Spec Pharm 2017, 23(3-b Suppl):S17-S24. 10.18553/jmcp.2017.23.3-b.s17 PMC10408422 28287347

[12] Richeldi L, du Bois RM, Raghu G, Azuma A, Brown KK, Costabel U, Cottin V, Flaherty KR, Hansell DM, Inoue Y (2014). Efficacy and safety of nintedanib in idiopathic pulmonary fibrosis. N Engl J Med.

[13] Kreuter M, Koegler H, Trampisch M, Geier S, Richeldi L (2019). Differing severities of acute exacerbations of idiopathic pulmonary fibrosis (IPF): insights from the INPULSIS(R) trials. Respir Res.

[14] Flaherty KR, Andrei AC, King TE, Raghu G, Colby TV, Wells A, Bassily N, Brown K, du Bois R, Flint A (2007). Idiopathic interstitial pneumonia: do community and academic physicians agree on diagnosis?. Am J Respir Crit Care Med.

[15] Lamas DJ, Kawut SM, Bagiella E, Philip N, Arcasoy SM, Lederer DJ (2011). Delayed access and survival in idiopathic pulmonary fibrosis: a cohort study. Am J Respir Crit Care Med.

[16] Raghu G, Anstrom KJ, King TE, Lasky JA, Martinez FJ, Idiopathic Pulmonary Fibrosis Clinical Research N (2012). Prednisone, azathioprine, and N-acetylcysteine for pulmonary fibrosis. N Engl J Med.

[17] HCUP Quality control procedures. Healthcare Cost and Utilization Project (HCUP) [www.hcup-us.ahrq.gov/db/quality.jsp]. Accessed 14 April 2020.

[18] Vu A, Vasireddy A, Moua T, Baqir M, Ryu JH. Clarifying the diagnosis of post-inflammatory pulmonary fibrosis: a population-based study. Eur Respir J. 2019;54(1):1900103. 10.1183/13993003.00103-2019 30956211

[19] Clinical Classifications Software Refined (CCSR) for ICD-10-CM Diagnoses. Healthcare Cost and Utilization Project (HCUP) [www.hcup-us.ahrq.gov/toolssoftware/ccsr/ccs_refined.jsp] . Accessed 14 April 2020.

[20] HCUP Clinical classifications software (CCS) for ICD-9-CM. Healthcare Cost and Utilization Project (HCUP) [www.hcup-us.ahrq.gov/toolssoftware/ccs/ccs.jsp] . Accessed 14 April 2020.

[21] HCUP Clinical classifications software for services and procedures. Healthcare Cost and Utilization Project (HCUP) [https://www.hcup-us.ahrq.gov/toolssoftware/ccs_svcsproc/ccssvcproc.jsp] . Accessed 14 April 2020.

[22] HCUP Comorbidity software. Healthcare Cost and Utilization Project (HCUP) [https://www.hcup-us.ahrq.gov/toolssoftware/comorbidity/comorbidity.jsp]. Accessed 14 April 2020.

[23] Caminati A, Lonati C, Cassandro R, Elia D, Pelosi G, Torre O, Zompatori M, Uslenghi E, Harari S. Comorbidities in idiopathic pulmonary fibrosis: an underestimated issue. Eur Respir Rev. 2019;28(153):190044. 10.1183/16000617.0044-2019 PMC9488913 31578211

[24] Kreuter M, Ehlers-Tenenbaum S, Palmowski K, Bruhwyler J, Oltmanns U, Muley T, Heussel CP, Warth A, Kolb M, Herth FJ (2016). Impact of comorbidities on mortality in patients with idiopathic pulmonary fibrosis. PLoS One.

[25] Oldham JM, Collard HR (2017). Comorbid conditions in idiopathic pulmonary fibrosis: recognition and management. Front Med (Lausanne).

[26] Tabachnick BG, Fidell LS (2013). Using multivariate statistics.

[27] Hutchinson J, Fogarty A, Hubbard R, McKeever T (2015). Global incidence and mortality of idiopathic pulmonary fibrosis: a systematic review. Eur Respir J.

[28] Raghu G, Chen SY, Yeh WS, Maroni B, Li Q, Lee YC, Collard HR (2014). Idiopathic pulmonary fibrosis in US Medicare beneficiaries aged 65 years and older: incidence, prevalence, and survival, 2001-11. Lancet Respir Med.

[29] Rush B, Wiskar K, Berger L, Griesdale D (2016). The use of mechanical ventilation in patients with idiopathic pulmonary fibrosis in the United States: a nationwide retrospective cohort analysis. Respir Med.

[30] Durheim MT, Judy J, Bender S, Baumer D, Lucas J, Robinson SB, Mohamedaly O, Shah BR, Leonard T, Conoscenti CS (2019). In-hospital mortality in patients with idiopathic pulmonary fibrosis: a US cohort study. Lung.

[31] Dove EP, Olson AL, Glassberg MK (2019). Trends in idiopathic pulmonary fibrosis-related mortality in the United States: 2000-2017. Am J Respir Crit Care Med.

[32] Fernández Pérez ER. Changing trends in age-adjusted pulmonary fibrosis mortality in the USA: a joinpoint regression analysis. Eur Respir J. 2019;54(1):1900364. 10.1183/13993003.00364-2019 31023853

[33] Ho ATN, Shmelev A, Charbek E (2020). Trends and seasonal variation of hospitalization and mortality of interstitial lung disease in the United States from 2006 to 2016. Respir Res.

[34] Raghu G, Rochwerg B, Zhang Y, Garcia CA, Azuma A, Behr J, Brozek JL, Collard HR, Cunningham W, Homma S (2015). An Official ATS/ERS/JRS/ALAT Clinical Practice Guideline: Treatment of Idiopathic Pulmonary Fibrosis. An Update of the 2011 Clinical Practice Guideline. Am J Respir Crit Care Med.

[35] Richeldi L, Launders N, Martinez F, et al. The characterisation of interstitial lung disease multidisciplinary team meetings: a global study. ERJ Open Res. 2019;5(2):00209–2018. 10.1183/23120541.00209-2018 PMC6441673 30949489

[36] Mooney JJ, Raimundo K, Chang E, Broder MS (2017). Mechanical ventilation in idiopathic pulmonary fibrosis: a nationwide analysis of ventilator use, outcomes, and resource burden. BMC Pulm Med.

[37] Karkkainen M, Kettunen HP, Nurmi H, Selander T, Purokivi M, Kaarteenaho R (2017). Effect of smoking and comorbidities on survival in idiopathic pulmonary fibrosis. Respir Res.

[38] King TE, Bradford WZ, Castro-Bernardini S, Fagan EA, Glaspole I, Glassberg MK, Gorina E, Hopkins PM, Kardatzke D, Lancaster L (2014). A phase 3 trial of pirfenidone in patients with idiopathic pulmonary fibrosis. N Engl J Med.

[39] Ley B, Collard HR, King TE (2011). Clinical course and prediction of survival in idiopathic pulmonary fibrosis. Am J Respir Crit Care Med.

[40] Adegunsoye A, Oldham JM, Bellam SK, et al. African-American race and mortality in interstitial lung disease: a multicentre propensity-matched analysis. Eur Respir J. 2018;51(6):1800255. 10.1183/13993003.00255-2018 PMC6089357 29724923

[41] Yamazaki R, Nishiyama O, Sano H, Iwanaga T, Higashimoto Y, Kume H, Tohda Y (2016). Clinical features and outcomes of IPF patients hospitalized for pulmonary infection: a Japanese cohort study. PLoS One.

[42] Pugashetti J, Graham J, Boctor N, Mendez C, Foster E, Juarez M, Harper R, Morrissey B, Kadoch M, Oldham JM. Weight loss as a predictor of mortality in patients with interstitial lung disease. Eur Respir J. 2018;52(3):1801289. 10.1183/13993003.01289-2018 PMC6643265 30072505

[43] Sprunger DB, Olson AL, Huie TJ, Fernandez-Perez ER, Fischer A, Solomon JJ, Brown KK, Swigris JJ (2012). Pulmonary fibrosis is associated with an elevated risk of thromboembolic disease. Eur Respir J.

[44] Alakhras M, Decker PA, Nadrous HF, Collazo-Clavell M, Ryu JH (2007). Body mass index and mortality in patients with idiopathic pulmonary fibrosis. Chest.

[45] Hyldgaard C, Hilberg O, Bendstrup E (2014). How does comorbidity influence survival in idiopathic pulmonary fibrosis?. Respir Med.

[46] Kolilekas L, Manali E, Vlami KA, Lyberopoulos P, Triantafillidou C, Kagouridis K, Baou K, Gyftopoulos S, Vougas KN, Karakatsani A (2013). Sleep oxygen desaturation predicts survival in idiopathic pulmonary fibrosis. J Clin Sleep Med.

[47] Kaunisto J, Kelloniemi K, Sutinen E, Hodgson U, Piilonen A, Kaarteenaho R, Makitaro R, Purokivi M, Lappi-Blanco E, Saarelainen S (2015). Re-evaluation of diagnostic parameters is crucial for obtaining accurate data on idiopathic pulmonary fibrosis. BMC Pulm Med.

[48] Wu X, Kim GH, Salisbury ML, Barber D, Bartholmai BJ, Brown KK, Conoscenti CS, De Backer J, Flaherty KR, Gruden JF, Hoffman EA, Humphries SM, Jacob J, Maher TM, Raghu G, Richeldi L, Ross BD, Schlenker-Herceg R, Sverzellati N, Wells AU, Martinez FJ, Lynch DA, Goldin J, Walsh SLF. Computed Tomographic Biomarkers in Idiopathic Pulmonary Fibrosis. The Future of Quantitative Analysis. Am J Respir Crit Care Med. 2019;199(1):12–21. 10.1164/rccm.201803-0444PP 29986154

[49] Lancaster L, Crestani B, Hernandez P, Inoue Y, Wachtlin D, Loaiza L, Quaresma M, Stowasser S, Richeldi L (2019). Safety and survival data in patients with idiopathic pulmonary fibrosis treated with nintedanib: pooled data from six clinical trials. BMJ Open Respir Res.

