# Supplementary material for: In-hospital mortality trends among patients with idiopathic pulmonary fibrosis in the United States between 2013-2017: a comparison of academic and non-academic programs
Source: BMC Pulm Med. 2020 Nov 7;20:289. doi: 10.1186/s12890-020-01328-y (PMC7648951; doi:10.1186/s12890-020-01328-y)
Supplement: Supplementary file 1 — Additional file 1. [file 12890_2020_1328_MOESM1_ESM.docx]

|  | **ICD-10 codes** | **ICD-9 codes** |
| --- | --- | --- |
| Idiopathic pulmonary fibrosis | J84.112 | 516.31 |
| Idiopathic nonspecific interstitial pneumonia | J84.113 | 516.32 |
| Acute interstitial pneumonia | J84.114 | 516.33 |
| Respiratory bronchiolitis–interstitial lung disease | J84.115 | 516.34 |
| Cryptogenic organizing pneumonia | J84.116 | 516.36 |
| Desquamative interstitial pneumonia | J84.117 | 516.37 |
| Nonspecific codes for interstitial lung diseases | J84.9 | 515 |
|  | J84.09 | 516.9 |
|  | J84.10 | 516.8 |
|  | J84.111 | 516.30 |
|  | J84.89 |  |

**Supplementary Materials**

**Table E1** Major idiopathic interstitial pneumonias and their international Classification of Diseases (ICD) codes for ninth and tenth editions that are abstracted in this study.

| Diagnoses abstracted using Elixhauser Comorbidity Software | Obesity, CHF, Pulmonary circulation disease, Chronic renal disease, Liver disease, Solid tumor w/o metastasis, Metastatic cancer, Rheumatoid arthritis, Diabetes mellitus and Hypothyroidism. |
| --- | --- |

**Table E2** Diagnoses abstracted using Elixhauser Comorbidity Software.(1)

**Table E3** International Classification of Diseases (ICD) codes for ninth and tenth editions that are abstracted in this study.

| **Diagnoses** | **ICD 10 codes** | | | **ICD 9 codes** | | |
| --- | --- | --- | --- | --- | --- | --- |
| Smoking: |  |  |  |  |  |  |
| Current | F17.200 | F17.201 | F17.203 | 305.1 |  |  |
|  | F17.208 | F17.209 | F17.210 |  |  |  |
|  | F17.211 | F17.213 | F17.218 |  |  |  |
|  | F1.7219 |  |  |  |  |  |
| Previous | Z87.891 |  |  | V15.82 |  |  |
| Dependence on long-term oxygen | Z99.81 |  |  | V46.2 |  |  |
| Respiratory failure: |  |  |  |  |  |  |
| Acute respiratory failure | J96.00 | J96.01 | J96.02 | 518.81 |  |  |
| Acute on Chronic respiratory failure | J96.20 | J96.21 | J96.22 | 518.84 |  |  |
| Chronic respiratory failure | J96.10 | J96.11 | J96.12 | 518.83 |  |  |
| Unspecified respiratory failure | J96.90 | J96.91 | J96.92 |  |  |  |
| Chronic obstructive lung disease | J44.0 | J44.1 | J44.9 | 491.20 | 491.21 | 491.22 |
| Obstructive sleep apnea | G47.33 |  |  | 327.23 |  |  |
| Gastroesophageal reflux disease | K21.0 | K21.9 |  | 530.11 | 530.81 |  |
| Frailty | R54 |  |  | 797 |  |  |
| Low body mass index (<20) | Z68.1 |  |  | V85.0 |  |  |
| New pulmonary embolism | I26.01 | I26.02 | I26.09 | 415.12 | 415.13 | 415.19 |
|  | I26.90 | I26.92 | I26.93 |  |  |  |
|  | I26.94 | I26.99 |  |  |  |  |
| Any history of lung transplant | Z94.2 | Z94.3 | Z48.24 | V42.6 |  |  |
|  | Z48.280 |  |  |  |  |  |
| **Procedures** |  |  |  |  |  |  |
| Lung transplant | 0BYC0Z0 | 0BYC0Z1 | 0BYC0Z2 | 33.50 | 33.51 | 33.52 |
|  | 0BYD0Z0 | 0BYD0Z1 | 0BYD0Z2 | 33.6 |  |  |
|  | 0BYF0Z0 | 0BYF0Z1 | 0BYF0Z2 |  |  |  |
|  | 0BYG0Z0 | 0BYG0Z1 | 0BYG0Z2 |  |  |  |
|  | 0BYH0Z0 | 0BYH0Z1 | 0BYH0Z2 |  |  |  |
|  | 0BYJ0Z0 | 0BYJ0Z1 | 0BYJ0Z2 |  |  |  |
|  | 0BYK0Z0 | 0BYK0Z1 | 0BYK0Z2 |  |  |  |
|  | 0BYL0Z0 | 0BYL0Z1 | 0BYL0Z2 |  |  |  |
|  | 0BYM0Z0 | 0BYM0Z1 | 0BYM0Z2 |  |  |  |

|  | **Clinical Classification System Refined (CCSR) for ICD-10 codes** | **Clinical Classification Software for ICD-9 codes** |
| --- | --- | --- |
| Pneumonia | RSP002 | 122 |
| Asthma | RSP009 | 128 |
| Rheumatoid arthritis and related disease | MUS003 | 202 |
| Systemic lupus erythematosus and connective tissue disorders | MUS024 | 210 |
| Lung disease due to external agents | RSP013 | 132 |
| **Procedures:** |  | |
| Mechanical ventilation (respiratory intubation) | 216 | |
| Diagnostic bronchoscopy and biopsy of bronchus | 37 | |

**Table E4** Procedures and diagnoses categories abstracted using clinical classification system tools for ICD-9 and ICD-10 (for procedures and diagnoses).(2-4)

The Clinical Classifications Software Refined (CCSR) for ICD-10-CM and the Clinical Classifications Software (CCS) for ICD-9-CM are software that categorize ICD diagnosis codes into a manageable number of clinically meaningful categories. It aggregates over 70,000 ICD-10-CM diagnosis codes and over 14,000 ICD-9-CM diagnosis codes.(2, 3) CCS-Services and Procedures provides a method for classifying Current Procedural Terminology (CPT®) codes and Healthcare Common Procedure Coding System (HCPCS) codes into clinically meaningful procedure categories. It aggregates more than 9,000 CPT/HCPCS codes and 6,000 HCPCS codes are collapsed into 244 clinically meaningful categories.(4)

| **Patient population** | **2013** | **2014** | **2015** | **2016** | **2017** | **P-value*** |
| --- | --- | --- | --- | --- | --- | --- |
| (a) Temporal trend in all-cause mortality in all IPF patients. | | | | | | |
| All-cause mortality in all IPF patients, n (%) | 1690 (10.6) | 1945 (11.6) | 2075 (11) | 2095 (10.7) | 2400 (10.6) | P=0.12 |
| (b) Temporal trends in mortality stratified by hospital academic status in all IPF patients and different subgroups. | | | | | | |
| Mortality in IPF patients hospitalized in academic institution, n (%) | 945 (12.1) | 1285 (12.4) | 1420 (12.1) | 1410 (10.9) | 1710 (10.9) | P<0.001 |
| Mortality in IPF patients hospitalized in non-academic institution, n (%) | 745 (9.2) | 660 (10.2) | 655 (9.2) | 685 (10.4) | 690 (9.9) | P=0.18 |
| Mortality in IPF patients with respiratory failure hospitalized in academic institution, n (%) | 840 (25.2) | 1165 (23.5) | 1290 (20.9) | 1285 (16.4) | 1585 (16.4) | P<0.001 |
| Mortality in IPF patients with respiratory failure hospitalized in non-academic institution, n (%) | 585 (17.9) | 545 (17.4) | 600 (16) | 610 (15.1) | 630 (14.3) | P<0.001 |
| Mechanical ventilation associated mortality in IPF patients hospitalized in academic institution, n (%) | 620 (45.9) | 865 (44.8) | 815 (39.4) | 770 (38.9) | 1030 (40.1) | P<0.001 |
| Mechanical ventilation associated mortality in IPF patients hospitalized in non-academic institution, n (%) | 355 (31.8) | 320 (34.4) | 415 (39.9) | 380 (37.4) | 365 (35.3) | P=0.03 |
| (c) Temporal trends in mortality stratified by the presence of respiratory failure. | | | | | | |
| Mortality in IPF patients with any respiratory failure, n (%) | 1425 (21.6) | 1710 (21.1) | 1890 (19.1) | 1895 (16) | 2215 (15.7) | P<0.001 |
| Mortality in IPF patients without respiratory failure, n (%) | 265 (2.9) | 235 (2.7) | 185 (2.1) | 200 (2.6) | 185 (2.2) | P=0.003 |
| (d) Temporal trends in mechanical ventilation associated mortality in all IPF patients, and stratified by presence of respiratory failure. | | | | | | |
| Mechanical ventilation associated mortality in all IPF patients, n (%) | 975 (39.6) | 1185 (41.4) | 1230 (39.5) | 1150 (38.4) | 1395 (38.7) | P=0.08 |
| Mechanical ventilation associated mortality in IPF patients with any respiratory failure, n (%) | 910 (43.1) | 1110 (44.2) | 1190 (42.8) | 1115 (40) | 1335 (39.3) | P<0.001 |
| Mechanical ventilation associated mortality in IPF patients without respiratory failure, n (%) | 65 (18.3) | 75 (21.4) | 40 (12.1) | 35 (16.7) | 60 (29.3) | P=0.1 |
| (e) Temporal trends in all-cause mortality stratified by age group. | | | | | | |
| Mortality in IPF patients (50-59) years old, n (%) | 255 (15.2) | 170 (10.6) | 190 (11.5) | 135 (9.2) | 185 (11.9) | P=0.001 |
| Mortality in IPF patients (60-69) years old, n (%) | 340 (9.9) | 465 (12.4) | 480 (11.8) | 435 (11) | 505 (11.2) | P=0.59 |
| Mortality in IPF patients (70-79) years old, n (%) | 515 (10.7) | 625 (11.9) | 710 (11.6) | 740 (10.6) | 845 (10.4) | P=0.08 |
| Mortality in IPF patients (≥80) years old, n (%) | 580 (9.8) | 685 (11.1) | 695 (9.9) | 785 (11) | 865 (10.2) | P=0.62 |
| (f) Temporal trend of mechanical ventilation therapy rate in IPF patients stratified by presence of respiratory failure. | | | | | | |
| mechanical ventilation therapy treatment in IPF patients with respiratory failure, n (%) | 2110 (31.9) | 2510 (31) | 2780 (28) | 2785 (23.5) | 3400 (24.1) | P<0.001 |
| mechanical ventilation therapy treatment in IPF patients without respiratory failure, n (%) | 355 (3.8) | 350 (4) | 330 (3.7) | 210 (2.7) | 205 (2.4) | P<0.001 |

**Table E5.** Temporal trends of mortality in the whole cohort and different subgroups, 2013-2017.

(a) All-cause mortality in all IPF patients; (b) Mortality stratified by hospital academic status in all IPF patients and different subgroups; (c) Mortality stratified by the presence of respiratory failure; (d) Mechanical ventilation associated mortality in all IPF patients, and stratified by presence of respiratory failure; (e) All-cause mortality stratified by age group; (f) Mechanical ventilation therapy rate in IPF patients stratified by presence of respiratory failure. (IPF: Idiopathic pulmonary fibrosis)

* P-trend using Cochran-Armitage test of trend.

**References:**

1. HCUP Comorbidity Software. Healthcare Cost and Utilization Project (HCUP). 2008 [cited 2020 4/14]. Available from: <https://www.hcup-us.ahrq.gov/toolssoftware/comorbidity/comorbidity.jsp>.

2. Clinical Classifications Software Refined (CCSR) for ICD-10-CM Diagnoses. Healthcare Cost and Utilization Project (HCUP). February 2020 [cited 2020 4/14]. Available from: <www.hcup-us.ahrq.gov/toolssoftware/ccsr/ccs_refined.jsp>.

3. HCUP Clinical Classifications Software (CCS) for ICD-9-CM. Healthcare Cost and Utilization Project (HCUP). 2006-2009 [cited 2020 4/14]. Available from: <www.hcup-us.ahrq.gov/toolssoftware/ccs/ccs.jsp>.

4. HCUP Clinical Classifications Software for Services and Procedures. Healthcare Cost and Utilization Project (HCUP). 2008 [cited 2020 4/14]. Available from: <https://www.hcup-us.ahrq.gov/toolssoftware/ccs_svcsproc/ccssvcproc.jsp>.

STROBE Statement—Checklist of items that should be included in reports of ***cohort studies***

|  | Item No | Recommendation | Page  No. | Relevant text from manuscript |
| --- | --- | --- | --- | --- |
| **Title and abstract** | 1 | (*a*) Indicate the study’s design with a commonly used term in the title or the abstract | 1-2 |  |
|  |  | (*b*) Provide in the abstract an informative and balanced summary of what was done and what was found | 1-2 |  |
| Introduction | | |  |  |
| Background/rationale | 2 | Explain the scientific background and rationale for the investigation being reported | 3-4 |  |
| Objectives | 3 | State specific objectives, including any prespecified hypotheses | 3-4 |  |
| Methods | | |  |  |
| Study design | 4 | Present key elements of study design early in the paper | 4 |  |
| Setting | 5 | Describe the setting, locations, and relevant dates, including periods of recruitment, exposure, follow-up, and data collection | 4-5 |  |
| Participants | 6 | (*a*) Give the eligibility criteria, and the sources and methods of selection of participants. Describe methods of follow-up | 5-6 |  |
|  |  | (*b*) For matched studies, give matching criteria and number of exposed and unexposed | N/A |  |
| Variables | 7 | Clearly define all outcomes, exposures, predictors, potential confounders, and effect modifiers. Give diagnostic criteria, if applicable | 4-6 |  |
| Data sources/ measurement | 8* | For each variable of interest, give sources of data and details of methods of assessment (measurement). Describe comparability of assessment methods if there is more than one group | 4-5 |  |
| Bias | 9 | Describe any efforts to address potential sources of bias | 4-5 | Methods: Data source |
| Study size | 10 | Explain how the study size was arrived at | 6-7 | Figure.1 |
| Quantitative variables | 11 | Explain how quantitative variables were handled in the analyses. If applicable, describe which groupings were chosen and why | 6 | Methods: statistical analysis |
| Statistical methods | 12 | (*a*) Describe all statistical methods, including those used to control for confounding | 6 |  |
|  |  | (*b*) Describe any methods used to examine subgroups and interactions | 6 |  |
|  |  | (*c*) Explain how missing data were addressed | 4,6 | Figure.1 |
|  |  | (*d*) If applicable, explain how loss to follow-up was addressed | N/A |  |
|  |  | (*e*) Describe any sensitivity analyses | none |  |
| Results | | |  |  |
| Participants | 13* | (a) Report numbers of individuals at each stage of study—eg numbers potentially eligible, examined for eligibility, confirmed eligible, included in the study, completing follow-up, and analysed | 7 | Figure.1 |
|  |  | (b) Give reasons for non-participation at each stage | 7 | Figure.1 |
|  |  | (c) Consider use of a flow diagram | 7 | Figure.1 |
| Descriptive data | 14* | (a) Give characteristics of study participants (eg demographic, clinical, social) and information on exposures and potential confounders | 7 | Table.1 |
|  |  | (b) Indicate number of participants with missing data for each variable of interest | 7 | Figure.1 |
|  |  | (c) Summarise follow-up time (eg, average and total amount) | N/A |  |
| Outcome data | 15* | Report numbers of outcome events or summary measures over time | 8 | Figure. 2-4, Table. E1, Figure E1&E2 |
| Main results | 16 | (*a*) Give unadjusted estimates and, if applicable, confounder-adjusted estimates and their precision (eg, 95% confidence interval). Make clear which confounders were adjusted for and why they were included | 9 | Table.2 |
|  |  | (*b*) Report category boundaries when continuous variables were categorized | 8 | Table E1e and Figure E1 |
|  |  | (*c*) If relevant, consider translating estimates of relative risk into absolute risk for a meaningful time period | N/A |  |
| Other analyses | 17 | Report other analyses done—eg analyses of subgroups and interactions, and sensitivity analyses | 8-9 |  |
| Discussion | | |  |  |
| Key results | 18 | Summarise key results with reference to study objectives | 9 |  |
| Limitations | 19 | Discuss limitations of the study, taking into account sources of potential bias or imprecision. Discuss both direction and magnitude of any potential bias | 12 |  |
| Interpretation | 20 | Give a cautious overall interpretation of results considering objectives, limitations, multiplicity of analyses, results from similar studies, and other relevant evidence | 9-12 |  |
| Generalisability | 21 | Discuss the generalisability (external validity) of the study results | 13 |  |
| Other information | | |  |  |
| Funding | 22 | Give the source of funding and the role of the funders for the present study and, if applicable, for the original study on which the present article is based | N/A |  |

*Give information separately for exposed and unexposed groups.

**Note:** An Explanation and Elaboration article discusses each checklist item and gives methodological background and published examples of transparent reporting. The STROBE checklist is best used in conjunction with this article (freely available on the Web sites of PLoS Medicine at http://www.plosmedicine.org/, Annals of Internal Medicine at http://www.annals.org/, and Epidemiology at http://www.epidem.com/). Information on the STROBE Initiative is available at http://www.strobe-statement.org.
