# Supplementary material for: In-hospital mortality trends among patients with idiopathic pulmonary fibrosis in the United States between 2013-2017: a comparison of academic and non-academic programs
Source: BMC Pulm Med. 2020 Nov 7;20:289. doi: 10.1186/s12890-020-01328-y (PMC7648951; doi:10.1186/s12890-020-01328-y)
Supplement: Supplementary file 2 — Additional file 2 : Figure E1. Temporal trends of all-cause mortality stratified by age group. Figure E2. Temporal trends of MV therapy rate stratified by presence of respiratory failure. Figure E3. Receiver operator curve for the regression model. [file 12890_2020_1328_MOESM2_ESM.pptx]

## Slide 1
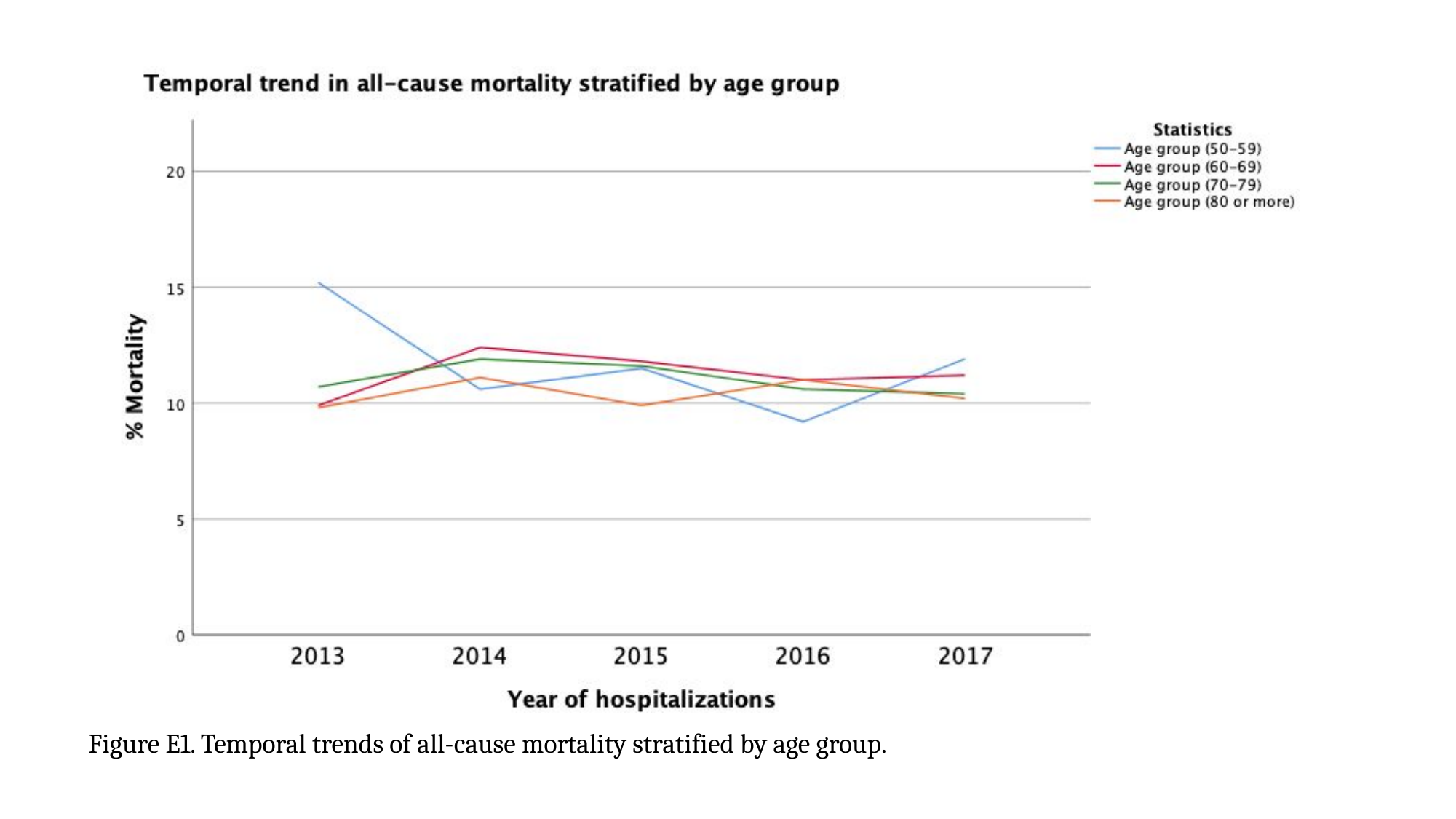

Figure E1. Temporal trends of all-cause mortality stratified by age group.

## Slide 2
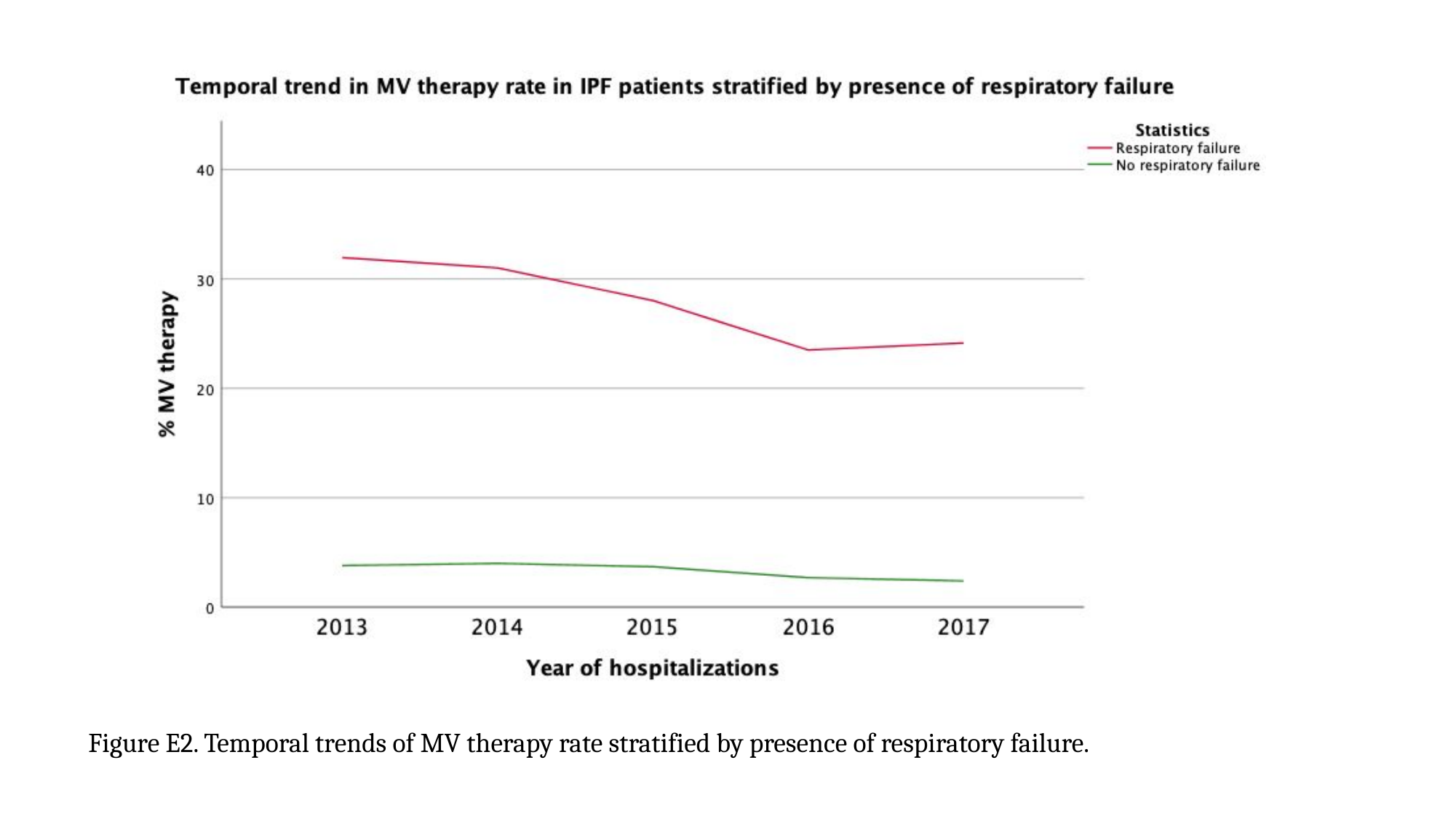

Figure E2. Temporal trends of MV therapy rate stratified by presence of respiratory failure.

## Slide 3
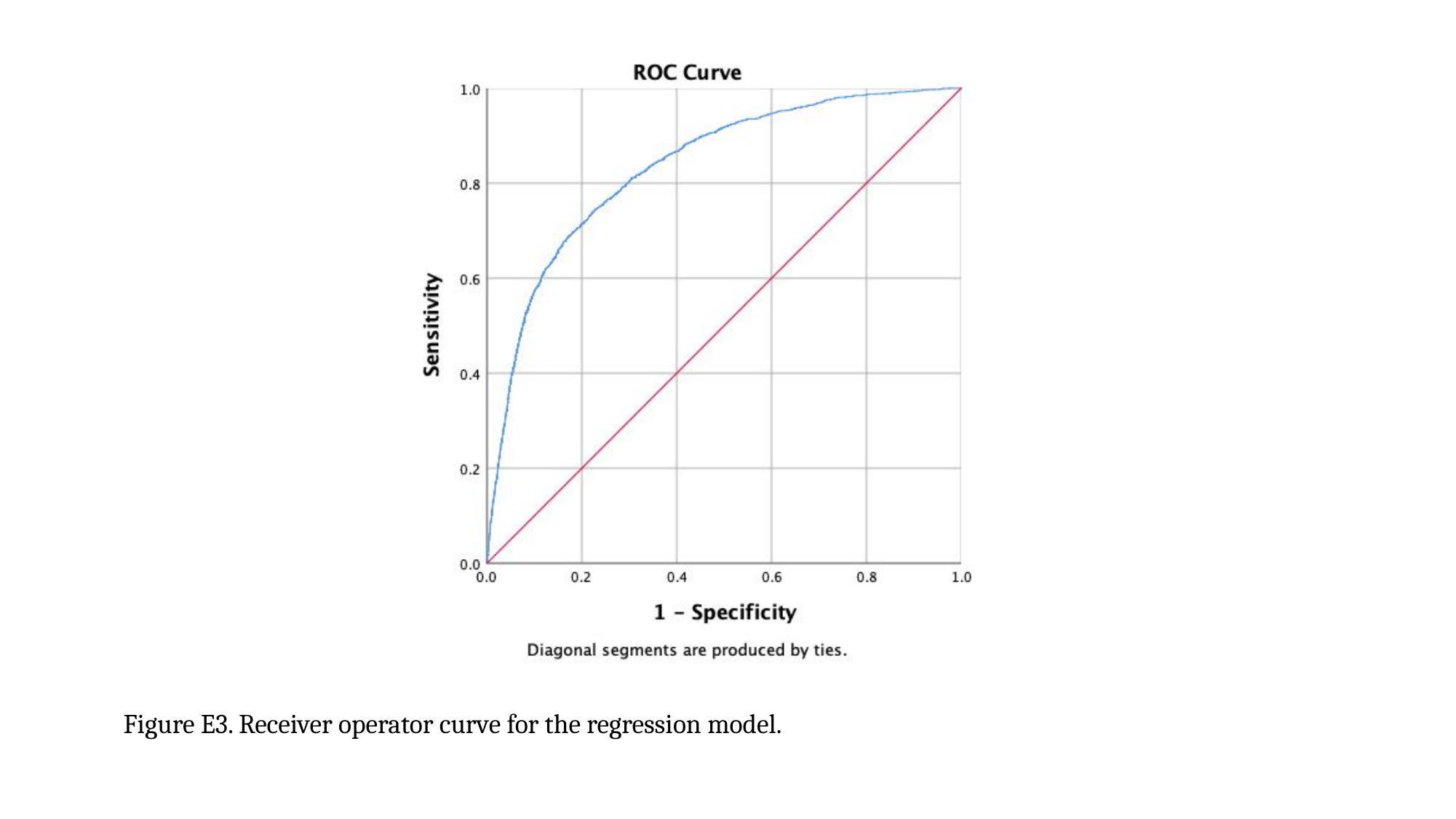

Figure E3. Receiver operator curve for the regression model.
